# Maternal and child health data quality in health care facilities at the Cape Coast Metropolis, Ghana

**DOI:** 10.1186/s12913-022-08449-6

**Published:** 2022-08-30

**Authors:** Obed Uwumbornyi Lasim, Edward Wilson Ansah, Daniel Apaak

**Affiliations:** Department of Health, Physical Education & Recreation, Faculty of Science and Technology Education, College of Education Studies, University of Cape Coast, Cape Coast, Ghana

**Keywords:** Data quality, Maternal and child health, Variables, Facilities, Accuracy, Timeliness, Completeness, Consistency, Verification factor

## Abstract

**Background:**

The demand for quality maternal and child health (MCH) data is critical for tracking progress towards attainment of the Sustainable Development Goal 3. However, MCH cannot be adequately monitored where health data are inaccurate, incomplete, untimely, or inconsistent. Thus, this study assessed the level of MCH data quality.

**Method:**

A facility-based cross-sectional study design was adopted, including a review of MCH service records. It was a stand-alone study involving 13 healthcare facilities of different levels that provided MCH services in the Cape Coast Metropolis. Data quality was assessed using the dimensions of accuracy, timeliness, completeness, and consistency. Health facilities registers were counted, collated, and compared with data on aggregate monthly forms, and a web-based data collation and reporting system, District Health Information System (DHIS2). The aggregate monthly forms were also compared with data in the DHIS2. Eight MCH variables were selected to assess data accuracy and consistency and two monthly reports were used to assess completeness and timeliness. Percentages and verification factor were estimated in the SPSS version 22 package.

**Results:**

Data accuracy were recorded between the data sources: Registers and Forms, 102.1% (95% CI = 97.5%—106.7%); Registers and DHIS2, 102.4% (95% CI = 94.4%—110.4%); and Forms and DHIS2, 100.1% (95% CI = 96.4%—103.9%). Across the eight MCH variables, data were 93.2% (95% CI = 82.9%—103.5%) complete in Registers, 91.0% (95% CI = 79.5%—102.5%) in the Forms, and 94.9% (95% CI = 89.9%—99.9%) in DHIS2 database. On the average, 87.2% (95% CI = 80.5%—93.9%) of the facilities submitted their Monthly Midwife’s Returns reports on time, and Monthly Vaccination Report was 94% (95% CI = 89.3%—97.3%). The overall average data consistency was 93% (95% CI = 84%—102%).

**Conclusion:**

Given the WHO standard for data quality, the level of MCH data quality in the health care facilities at the Cape Coast Metropolis, available through the DHIS2 is complete, reported on timely manner, consistent, and reflect accurately what exist in facility’s source document. Although there is evidence that data quality is good, there is still room for improvement in the quality of the data.

## Background

Health system data is increasing exponentially following United Nations’ 2030 agenda for sustainable development, with 17 Sustainable Development Goals (SDGs) and accompanying 232 indicators [1]. Particularly for populations with higher risks of disease and mortality, such as pregnant mothers, infants and children, the demand for high quality data is more crucial. The increase demands and production of data place pressure on national monitoring and reporting systems, especially in Low-and-Middle-Income Countries (LMICs) [2], necessitating the need for robust routine health information management practice in the provision of healthcare data [3, 4]. Data for decision making in healthcare are often generated by the health information systems, through the routine health information system (RHIS) [5]. The purpose of RHIS is to systematically collect quality data to effectively track and manage the needs and health status of the population and help decision-makers to allocate resources, plan, and prioritise services that will significantly impact the society [6, 7]. RHIS can also be used to assess disruptions during health emergencies, such as pandemics, which is important given the current context. It has increasingly been used to assess disruptions during COVID-19 over the past two years, and quality data is essential for this to be accurately assessed [8–10]. Despite the importance of RHIS, challenges of data quality including, accuracy, completeness, timeliness and consistency have been identified in many LMICs [4, 11–15]. This could jeopardise the effectiveness in achieving health targets both at the national and sub-national levels [16].

Accuracy of data is defined as how close data values are to the reality, or the truthfulness of the provided information [17]. It determines whether the data in the dataset is correct and exactly reflect what it should [18]. Data completeness refers to the level at which data includes items that are important to support the reason for which it was collected [18]. Timeliness defines the level at which a given set of data with respect to a specified time is current [18]. Consistency describes the extent to which data remain the same or identical [18]. Recent studies reported that facility-reported data were incomplete by 40% of the time in Nigeria [12] and between 32 to 75% from Ethiopia. For accuracy of data, previous studies found both under- and over-reporting of data varying across variables, facilities, and districts [12, 13]. For instance, under-reporting of 10–60% at facility level has been reported in Nigeria [12] and over-reporting for antenatal care-related data than for other variables from Rwanda [19]. In some cases, missing values, measurement error, inaccuracy and false reports from unidentified sources have been observed [20].

The primary data at health facilities in Ghana is mostly paper-based using registers, forms and notebooks. Subsequently, these data are collated and summarised to nationally standard designed forms and finally captured electronically into a database known as District Health Information System (DHIS2). At the facility level, primary sources of maternal data are captured into the maternal health record book (usually with the client), the antenatal register, delivery register, postnatal register, and the Expanded Programme on Immunisation (EPI) tally booklet that captures data on tetanus–diphtheria immunization for women, as well as Penta1 and Penta3 immunisation for children [11]. Often, the pregnant mother is assigned a unique identification number during registration and her details, including, biodata, parity, haemoglobin level, administration of tetanus–diphtheria, intermittent preventive treatment in pregnancy (IPT), etc. are captured onto the antenatal register. Moreover, deliveries services are recorded in a delivery register (sometimes labelled Returns on Delivery Book or Labour room admission and discharge book), and postnatal services recorded in the postnatal registers, with the clients’ biodata and other variables. Data on vaccination are captured into the vaccination tally sheet. At the end of the month, data from these sources at the facilities are collated and summarized- mostly by the midwives and community health nurses onto the monthly midwives returns form and monthly vaccination form. Before entries are made in the DHIS2 database, these summaries are reviewed by the head of the facility or validation team.

Over the years, efforts had been made in Ghana to improve the collection and management of health data at the national and sub-national levels. One of such efforts is the development of DHIS2 software. Notwithstanding the touted prospects of DHIS2 following its introduction as a “game changer” in better standardisation of data collection, leading to improvements in data quality, persistent data quality challenges continue to exist [21]. In practice, no health data regardless of its source can be considered perfect, they are subject to some quality limitations such as, human errors in data entry and computation, bias, missing values, and measurement errors [11]. Yet, high quality data is needed to monitor and evaluate programmes in LMICs striving towards universal health coverage. On the part of health care professionals, challenges in counting from registers and tally sheets, inability to understand the variables, problems in filling records, and inability to plot graphs to monitor progress and performance have been reported [22]. In the case of DHIS2, the data is collected in paper format (registers and standardized forms) at the facility level before it is transferred into the DHIS2 mostly at the sub-district and district level. Data quality assessments should therefore be undertaken to understand how much confidence can be placed in such data that are used to assess health sector performance and to understand the relative strengths and weaknesses of the data sources [4]. Therefore, this study assessed the level of MCH data quality at healthcare facilities in Cape Coast Metropolis focusing on the accuracy of data from the original source (facility register) to the final point (DHIS2), its completeness, timeliness, and consistency.

## Methods

A facility-based cross-sectional study design involving review of records of MCH service data was used.

The study was conducted in 13 healthcare facilities in the Cape Coast Metropolis—the only metropolis, out of the 22 districts in the Central Region of the Ghana. The Metropolis has fertility rate of 2.2 and a general fertility rate of 59.2 births per 1000 women aged 15–49 years [23]. In 2019, the metropolis had 38 health facilities of all types.

The sampling was done in two stages, one was the selection of the Cape Coast Metropolis out of the 22 districts in the region. The Metropolis was purposefully selected because of its uniqueness as one of the largest districts in the region and the only one with the full cadre of health facilities, including, a Teaching Hospitals. The second stage involved the selection of the health facilities. Desk review of documents showed 38 health facilities both government and private are situated in the Metropolis. Thirteen health facilities; 4 private and 9 government/public that met the inclusion criteria of providing MCH services in the Metropolis, were selected.

We relied on key variables for conducting MCH data quality assessment recommended by WHO [24]. Based on this recommendation, the MCH variables selected were, antenatal care first (ANC1) coverage, antenatal care first fourth (ANC4) coverage, first dose of intermittent preventive treatment in pregnancy (IPT1), administration of Tetanus–Diphtheria Vaccine (Td2 +) in pregnancy, deliveries attended by a skilled birth attendant/midwife in a health facility, access to early postnatal care (PNC), pentavalent vaccine first and third (Penta1 and Penta3) dose coverage in children under one year of age (Table 1).

**Table 1:** MCH variables with definition and data source

Variables	Definition	Data source
ANC1	Number of pregnant women reporting for antenatal care for the first time to any health facility with their current pregnancy	ANC register
ANC4	Number of pregnant women making their 4th antenatal visit for the period	ANC register
TD2 +	Number of pregnant women who have had two doses of Tetanus–Diphtheria (TD) for their current pregnancy OR require only one dose for their current pregnancy OR have completed their TD schedule and therefore do not require any dose for their current pregnancy	ANC register
IPT1	Number of pregnant women given their first dose of Sulfadoxine Pyrimethamine (SP) at ANC	ANC register
Deliveries	Total number of deliveries	Delivery register
PNC	Mothers accessing PNC for the first time after delivery	PNC register
Penta1	Number of children under 1 year receiving the Penta1 vaccine in the year	EPI returns
Penta3	Number of children under 1 year receiving the Penta3 vaccine in the year	EPI returns

Source: Ghana Health Service: Standard Operating Procedures for Health Information Managers, 2012

A data collation sheet was used to collect data. Three data sources were used to assess the routine data quality metrics: primary source data at health facilities (antenatal registers, delivery registers, postnatal registers, and EPI tally sheets); facility aggregate data (Midwife’s returns form and vaccination form); and facility-reported data in DHIS2. The ANC registers, PNC registers, Delivery book registers, and EPI tally book were used to collect data on accuracy of MCH variables. For each selected MCH variable, we recounted the data in the register on monthly basis and the results documented in a data collation sheet. Further, data in the monthly midwives, and vaccination report forms were documented in the data collation sheet for each of the selected variables. The same process was repeated for facility-reported data in DHIS2 for midwives returns report and vaccination report. The focus for data completeness, timeliness, and consistency was the data in DHIS2 database and not the registers or facility forms. Therefore, two main reports (the reporting rate summary and the summary reporting form) were extracted from DHIS2 database. The reporting rate summary was used to assess the completeness and timeliness of facility reporting, whereas summary reporting form assessed the completeness of indicator data and consistency of data (consistency over time, consistency between related data, and outliers in the referenced year). The reporting periods for data accuracy, timeliness, and completeness assessment were January 2020 to December, 2020, and that of consistency was January 2017 to December 2020. Consequently, a yearly report for the three years, (January 2017 to December 2019), was downloaded from the DHIS2 database to serve as comparison for assessing the consistency of data overtime.

A two-day training (with pre-test) was given to two research assistants (RAs with bachelor’s degree in information studies) who subsequently reviewed the documents. The first author did daily supervision to ensure that all collected data were complete and consistent among the two RA. There was largely agreement between the two RAs recounted data, except in one facility where variations were observed once in their figures for two variables (deliveries, and PNC). Subsequently, new collation sheets were given to the RAs to recount the data for the two variables, where the figures tallied.

### Data analysis

Data analysis was carried out using the Statistical Package for the Social Sciences (SPSS, version 22) for Windows. Frequencies and percentages and verification factors (VF) were calculated to characterise data quality by accuracy, completeness, timeliness and consistency.

#### Accuracy

**Fig. 1:**
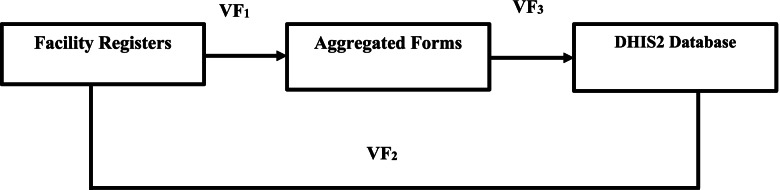
Verification factor from facility register to DHIS2 database

MCH data accuracy was determined through data accuracy checks, which involved verification of the numerical consistency of the recoded data in the (1) RHIS registers kept at the facility, (2) monthly aggregated form generated from the registers, and (3) data found in DHIS2 database, for the eight selected MCH variables, using VF. Verification factor is a summary indicator that measures the ratio of the number of recounted events from source documents to the number of reported events over the same period. Thus, VF is equal to the number of recounted data in the source document divided by the number of reported data in the forms or DHIS2 multiplied by 100. The mean and associated 95% confidence interval (95% CI) of each variable was calculated. When the value of the re-count data and variable data reported are equal, VF is equal to 1 and the report is said to be ideal. Any deviation from VF of 1 is indicative of either under (VF greater than 1) or over reporting (VF less than 1). The difference of an ideal reported VF and observed VF (1-VF) demonstrates either under-reporting or over-reporting. A report was considered accurate if the VF was within ± 10 precision (between 0.9 and 1.10), and inaccurate if the ratio of recount data to the reported data was less than 0.9 or greater than 1.10. Three types of VFs were calculated for data accuracy across the three data sources (registers, aggregated forms, and DHIS2 database). Verification factor 1 (VF_1_) measures the error in data transfer from the registers to the aggregate data forms; VF_2_ measures the error in data transfer from the registers to the DHIS2 database; and VF_3_ measures the error in transferring data from the aggregate form to the DHIS2 platform, as shown in Fig. 1 below.

#### Data completeness

Data completeness was assessed in two strands: completeness of the reports, and completeness of variable data reported in DHIS2. Two reports (Monthly Midwife’s Returns for maternal health variables, and Monthly Vaccination Report for child health variables) were considered for completeness of the reports. Facilities which submitted these two reports for the 12 months of 2020 into the DHIS2 platform were assessed. The ratio of total reports available/received to the total reports expected were calculated to show the level of completeness of the reports. Completeness of indicator data reported in DHIS2 was assessed by finding the ratio of number of reports that are complete to the total reports available/received.

#### Timeliness

Timeliness of facility reporting data into DHIS2 was assessed by finding the percentage of facility’s expected monthly reports against the actual reports submitted into the DHIS2 on or before a Ghana Health Service (GHS) scheduled date (5^th^ of the ensuing month) for Monthly Midwife’s returns report, and Monthly Vaccination Report.

#### Consistency

Consistency of the data was assessed under three groupings: consistency over time, consistency between related variables, and consistency of event reporting. Consistency over time was analysed by finding the mean ratio of an indicator for reference year (2020) to the mean of the same indicator for the three preceding years (2017, 2018, 2019) combined. Data was considered consistent over time if the reported value for the reference year is within ± 33% of the mean value for the preceding three years, taking into consideration any expected changes in the patterns of service delivery [24]. Consistency over time was also assessed to ascertain how individual facility’s values were consistent or different from the district values for the eight MCH data reported into DHIS2 database. Consistency of related variables was analysed by calculating the facility’s ratio for values of indicator-pairs that have a predictable relationship. The indicator pairs considered includes: Penta1 and ANC1; Penta1 and Penta3; and ANC1 and ANC4. Outlier analysis was used to assess consistency of event reporting. Two types of outliers (moderate and extreme) were calculated. Values that were at least two standard deviations from the average value for the MCH variable at a specified time were considered moderate and three standard deviations were considered extreme outliers.

## Results

Though we sampled 13 health care facilities, five facilities were not providing some of the services of the variables considered in this research and therefore had no data in DHIS2 for such variables. Thus, eight facilities that provided all the eight MCH variables were considered in the consistency over time analysis and the remaining five that did not provide any of the services in the previous years were dropped from this analysis. Also, one facility was not providing child health services, hence 12 facilities were used in assessing the completeness of vaccination report, and the consistency between variables.

### Data accuracy

#### Data accuracy between the registers and monthly reported forms

**Fig. 2:**
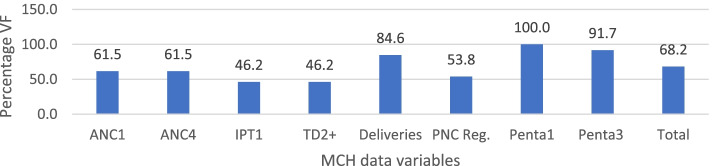
Percentage of facilities within 10% tolerance levels for accuracy between the registers and forms

An overall accuracy between the registers and forms at health facilities was 102.1% (95% CI = 97.5%—106.7%) with variations observed among the variables and months (Table 2). Four of the eight variables (ANC1, ANC4, Td2 + , and IPT1) had scores above 100%, suggesting under-reporting [24] of recounted data from the registers to the monthly report forms. Four of the variables (deliveries, PNC registrants, Penta1 and Penta3) had values below 100%, suggesting an over-reporting of the monthly report forms. Apart from February and March which recorded a VF of less than 100% (indicating under-reporting) and December which recorded VF of 100% (no variation), the rest of the months recorded over-reporting of data from the registers to the monthly report forms. The percentage of MCH data accuracy between the registers and forms were observed for the facilities (Fig. 2). For child health services, all the facilities were within the threshold recommended by WHO for data accuracy (± 10% tolerance limits) [24] for Penta1, whereas about 92% were within the set limits for Penta3. For the maternal health variables, 85% of the facilities were within the threshold for data accuracy for deliveries, 62% each for ANC1 and ANC4, 54% for PNC, and 46% each for IPT1 and Td2 + . About 68% of the facilities’ data were within the ± 10% tolerance limits for data accuracy for all the eight variables when data found in the registers were compared to that on the forms.

**Table 2:** Data accuracy between the Registers and Forms

Variables	Months												Statistics: Overall mean VFs				
	**Jan**	**Feb**	**Mar**	**Apr**	**May**	**Jun**	**Jul**	**Aug**	**Sep**	**Oct**	**Nov**	**Dec**	**Mean**	**SD**	**Min**	**Max**	**95% CI**
ANC1	109.0	105.3	106.6	102.8	106.5	107.2	108.6	102.5	**114.1**	106.4	105.6	106.8	106.7	3.1	102.5	114.1	1.92
ANC4	**129.4**	108.9	103.0	109.8	100.7	109.8	98.5	99.6	**113.8**	107.9	98.2	106.6	107.4	8.7	98.2	129.4	5.5
IPT1	105.6	96.1	96.3	98.1	**130.6**	105.7	97.4	99.7	100.9	94.4	103.9	98.2	101.3	9.7	94.4	130.6	6.15
Td2 +	**112.8**	99.1	99.7	108.3	**115.9**	100.5	**114.0**	101.2	105.0	105.7	**171.9**	**119.9**	**110.6**	19.8	99.1	171.9	12.61
Deliveries	105.3	99.3	101.0	98.4	97.1	100.0	100.2	101.1	98.0	100.4	97.5	100.2	99.7	2.2	97.1	105.3	1.38
PNC Reg	91.7	95.3	95.6	95.9	95.5	93.4	94.1	96.1	95.4	93.4	97.2	94.8	95.0	1.49	91.7	97.2	0.95
Penta1	89.8	96.0	97.0	97.0	99.7	99.1	100.9	**112.1**	**88.9**	103.8	**121.3**	89.9	99.3	9.4	88.9	121.3	5.99
Penta3	**87.8**	91.1	92.0	99.3	100.0	95.6	98.5	96.8	99.7	109.7	98.3	93.5	96.9	5.6	87.8	109.7	3.56
Mean	103.8	99.0	99.0	100.4	102.1	100.6	100.8	101.1	100.9	102.1	105.8	100.0	102.1	5.5	95	110.6	4.61

Values outside the threshold recommended by WHO for data accuracy (± 10% tolerance limits) boldened

#### Data accuracy between the registers and monthly report in DHIS2

**Fig. 3:**
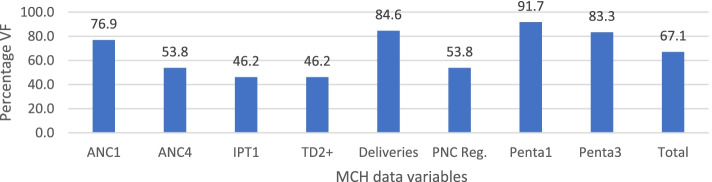
Percentage of facilities within 10% tolerance levels for data accuracy between the registers and DHIS2

Apart from January and November that had over-reporting, 92.5% and 97.9% respectively, the rest of the months saw under reporting from the registers to DHIS2 database (Table 3). In addition, inaccuracies were observed in January for five of the variables, three variables each in May and September, two variables each in June, August, and December, and one indicator each in February, March, April, July, and October, at the 100% ± 10%. An overall figure of 102.4% (95% CI = 94.4%—110.4%) data accuracy was recorded between data in the registers and DHIS2 (Table 3). For the child health variables, about 92% of the facilities were within the set limits for Penta1 and 83% for Penta3 (Fig. 3). For maternal health variables, about 85%, 80%, 54% and 46% facilities were within the ± 10% tolerance level for deliveries, ANC1, ANC4 and PNC, IPT1 and Td2 + , respectively (Fig. 3). Whereas the VF for government owned facilities was between 90 and 110% for all the MCH variables except for Td2 + , the privately owned facilities had two of the MCH variables (ANC1 and PNC) outside the acceptable WHO threshold for data accuracy. It was also observed that the Teaching Hospital, district hospitals and health centres had VFs within the limit (90% to 110%) for all the MCH variables. In contrast, clinics had VF of 66.4% and 75.5% for Community Health Planning and Services (CHPs) compounds for PNC, and Metropolitan hospital had 296.5% for Td2 + and 120.6% for Penta3 (Table 4).

**Table 3:** Data accuracy between Registers and DHIS2

Variables	Months												Statistics: Overall mean VFs				
	**Jan**	**Feb**	**Mar**	**Apr**	**May**	**Jun**	**Jul**	**Aug**	**Sep**	**Oct**	**Nov**	**Dec**	**Mean**	**SD**	**Min**	**Max**	**95%CI**
ANC1	108.6	105.3	105.6	103.0	106.8	107.5	106.0	103.1	**111.9**	103.5	109.3	106.4	106.4	2.7	103	111.9	1.69
ANC4	**127.0**	103.6	103.0	109.8	100.7	108.6	96.4	101.2	106.6	107.9	101.2	105.9	106.2	7.7	96.4	127	4.87
IPT1	105.0	95.9	96.0	97.8	**129.4**	109.2	97.7	97.6	98.6	93.3	109.0	95.9	101.1	10.1	93.3	129.4	6.39
Td2 +	108.2	**144.5**	**119.2**	149.2	**113.2**	**123.4**	**143.9**	**114.6**	**121.4**	**117.8**	99.6	**124.9**	**122.3**	15.3	99.6	149.2	9.7
Deliveries	**86.0**	99.3	101.0	100.5	97.2	99.5	99.7	101.5	96.9	99.4	98.7	100.2	98.4	4.1	86	101.5	2.61
PNC Reg	**56.0**	94.9	95.3	96.2	93.0	90.9	92.9	93.7	92.9	91.5	95.3	99.8	90.8	11.3	56	99.8	7.17
Penta1	**89.8**	96.9	97.0	97.0	100.0	93.0	100.9	**111.8**	92.5	100.3	**84.5**	94.4	96.4	6.8	84.5	111.8	4.29
Penta3	**88.7**	91.1	92.0	99.3	**119.6**	95.6	98.2	93.9	**111.4**	107.9	**84.6**	90.5	97.4	10.3	84.6	119.6	6.56
Mean	92.5	101.8	100.2	103.1	103.2	100.4	101.3	101.0	102.0	101.4	97.9	101.2	102.4	9.6	90.8	122.3	8.00

Values outside the threshold recommended by WHO for data accuracy (± 10% tolerance limits) boldened

**Table 4:** Data accuracy between registers and monthly report in DHIS2 by facility type and ownership

Indicator	By care						By ownership	
	**Teaching Hospital**	**Metro Hospital**	**District Hospitals**	**Clinics**	**Health Centres**	**CHPS Compound**	**Government**	**Private**
ANC1	106.2	100.0	**111.7**	109.8	106.2	107.3	104.4	**121.5**
ANC4	102.4	104.7	**128.2**	104.4	102.4	95.4	106.0	108.0
IPT1	100.2	100.0	96.4	100.9	100.2	97.8	101.4	98.4
Td2 +	109.8	**269.5**	100.6	95.9	109.8	103.3	**126.8**	91.9
Deliveries	100.3	100.4	100.6	100.0	100.3	90.6	98.5	95.9
PNC Reg	95.7	102.4	97.8	**66.4**	95.7	**75.5**	92.0	**76.0**
Penta1	96.2	100.2	102.7	97.4	96.2	96.4	96.4	97.0
Penta3	91.6	**120.6**	96.4	96.2	91.6	93.2	97.7	95.3

Values outside the threshold recommended by WHO for data accuracy (± 10% tolerance limits) boldened

#### Data accuracy between Forms and DHIS2

**Fig. 4:**
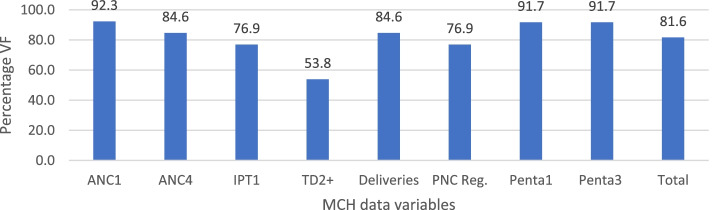
Percentage of facilities within 10% tolerance levels for data accuracy between the Form and DHIS2

Aside Td2 + and Penta3 that had VF above 100%, the rest of the variables had VF below 100% (Table 5). The overall data accuracy found in the monthly report and that of the DHIS2 database was 100.1% (95% CI = 96.4%—103.9%) (Table 5), indicating that the overall MCH data in DHIS2 were accurate. Further, about 31% of the facilities data were within the ± 10% tolerance limits for data accuracy for all the MCH variables. About 92% of the facilities were within the set limits for data accuracy for the child health variables (Penta1 and Penta3). Further, 92%, 85%, 77%, and 54% of the facilities were within the WHO recommendations threshold for the maternal health variables, ANC1, ANC4 and deliveries, IPT1 and PNC, and Td2 + respectively (Fig. 4).

**Table 5:** Data accuracy between Forms and DHIS2

Variables	Months													Statistics: Overall mean VFs				
	**Jan**	**Feb**	**Mar**	**Apr**	**May**	**Jun**	**Jul**	**Aug**	**Sep**	**Oct**	**Nov**	**Dec**	**Mean**		**SD**	**Min**	**Max**	**95%CI**
ANC1	99.6	100.0	99.1	100.3	100.3	100.2	97.6	100.7	98.1	97.3	103.5	99.6	99.7		1.6	97.3	103.5	1.04
ANC4	98.2	95.1	100.0	100.0	100.0	98.9	97.8	101.5	93.7	100.0	103.0	99.3	98.9		2.6	93.7	103	1.62
IPT1	99.4	99.7	99.7	99.7	99.1	103.3	100.4	97.9	97.7	98.8	104.9	97.7	99.8		2.9	97.7	104.9	1.39
Td2 +	95.9	**145.8**	**119.7**	**137.7**	97.7	**122.7**	**126.2**	**113.2**	**115.5**	**111.5**	**58.0**	104.1	**110.6**		22.6	58	145.8	14.38
Deliveries	**81.7**	100.0	100.0	102.2	100.2	99.5	99.5	100.4	98.9	99.0	101.3	100.0	98.7		5.4	81.7	102.2	3.42
PNC Reg	**61.1**	99.6	99.7	100.3	97.3	97.3	98.7	97.5	97.4	98.0	98.1	105.3	95.6		11.2	61.1	105.3	7.10
Penta1	100.0	100.9	100.0	100.0	100.3	93.8	100.0	99.7	104.0	96.6	**69.6**	105.1	97.1		9.3	69.6	105.1	5.88
Penta3	101.0	100.0	100.0	100.0	**119.6**	100.0	99.7	96.9	**111.7**	98.4	**86.0**	96.8	100.6		8.2	86	119.6	5.18
Mean	**89.1**	102.9	101.2	102.7	101.1	99.8	100.5	99.9	101.1	99.3	92.5	101.2	100.1		4.5	95.6	110.6	3.78

Values outside the threshold recommended by WHO for data accuracy (± 10% tolerance limits) boldened

### Completeness of MCH data

All (100%) the healthcare facilities submitted the two reports (Midwife’s Returns and Vaccination Report) for the 12 months of 2020 into the DHIS2 platform that reflected the monthly utilization of their MCH services, indicating a complete reporting rate.

Completeness of indicator data reported in DHIS2 was assessed by observing the zero or missing values for the eight MCH variables in DHIS2. We observed that facilities data in DHIS2 did not distinguish between missing values and true zero values. For example, a facility may have provided delivery services to clients but did not include this in their monthly report (missing value). Contrarily, a remote facility may have been equipped to provide delivery services but had no clients (for delivery) during a review month (true zero value). Both situations in the DHIS2 were presented as missing data. Generally, completeness was best for the child health variables, that is, 100% for Penta3 and 99.4% for Penta1, which indicate that all the data were entered into the DHIS2 (Table 6). Deliveries recorded the lowest completeness with 9% missing/zero values in DHIS2, followed by Td2 + (8.3%), IPT1 (7.7%), ANC4 (5.8%), PNC registrants (5.5%), and ANC1 (2.6%) as shown in Table 6. Overall, a 4.8% (95% CI = 1.5%—7.7%) zero or missing values was observed in DHIS2 for all the eight MCH variables.

**Table 6:** Zero or missing values by variables in DHIS2

Variables	# Missing values (Numerator)	# of values expected in the year (Denominator)	Numerator/Denominator
ANC1	4	156	2.6
ANC4	9	156	5.8
IPT1	12	156	7.7
Td2 +	13	156	8.3
Deliveries	14	156	9.0
PNC Reg	7	156	4.5
Penta1	1	144	0.7
Penta3	0	144	0.0
Total	60	1224	
**Mean**		**4.8**	
**Standard deviation**		**3.5**	
**95% CI**		± **2.9**	

From Table 7, MCH variables were 100% complete in the registers for all the levels of health care except in CHPs compounds where 78.4% was recorded. The metropolitan hospital had all variables 100% complete for the three data sources, whereas the CHPS compounds had less than 90% of all their MCH variables complete in all the three data sources. The private owned health facilities had an average of 84.5% completeness rate for all the three data sources, and a 96.8% completeness rate in the public sector facilities.

**Table 7:** Completeness of MCH indicators in the data sources by facility type

Data sources	By care					By ownership		
	Teaching Hospital	Metro Hospital	District Hospitals	Clinics	Health Centres	CHPS Compound	Government	Private
Registers	100	100	100	100	99.5	78.4	97.1	84.5
Forms	93.8	100	99	97.9	99	75	95.7	80.6
DHIS2	96.9	100	99	96.5	98.5	88.2	97.5	89.2
**Average**	**96.9**	**100.0**	**99.3**	**98.1**	**99.0**	**80.5**	**96.8**	**84.8**

### Timeliness of the report

Table 8 show the timeliness of facility report submitted on time. A 100% reporting rate on time was recorded in 38.5% of the facilities for Monthly Vaccination Report, and 15% for Monthly Midwife’s returns. Whereas 54% of the facilities recorded a 90 – 99% reporting rate on time for Monthly Midwife’s returns report, that of Monthly Vaccination report was 46%. On the average, 87.2% (95% CI = 80.5%—93.9%) of the facilities submitted their monthly Midwife's Returns reports to the next level on time, and that of Monthly Vaccination Report was about 94% (95% CI = 89.3%—97.3%).

**Table 8:** Reporting rate on time across the two monthly reports for health facilities

Timeliness range	Monthly Midwife’s Returns	Monthly Vaccination Report
	N (%)	N (%)
< 80%	3 (23.1)	0 (0)
80 – 89%	1 (7.7)	2 (15.4)
90 – 99%	7 (53.8)	6 (46.2)
100%	2 (15.4)	5 (38.5)
**Mean**	87.2	93.6
**Standard Deviation**	11.1	6.0
**Minimum**	66.7	83.3
**Maximum**	100	100
**9**5% C.I	± 6.70	± 3.70

### Consistency over time

The ratio for 2020 to the mean of the three preceding years (2017, 2018, 2019) for ANC1, ANC4, IPT1, Td2 + , Deliveries, PNC, Penta1 and Penta3 were respectively, 0.91, 0.90, 0.89, 0.79, 0.94, 1.16, 0.89, and 0.97 (Table 9). An overall average ratio of 0.93 (95% CI = 0.84 to 1.02) consistency over time was observed, suggesting an overall 7% decrease in the MCH service outputs for 2020 when compared with that of the preceding three years across the eight variables. Further, consistency over time was assessed at the facility level to ascertain how individual facility’s values were consistent or differ from the district values for the eight MCH variables data reported into DHIS2 database (Table 9). Estimate of consistency over time at the facility level showed that about 88% of the facilities recorded more than 33% difference between their ratio and the district ratio for at least one of the eight MCH variables (Table 9). The percentage difference between the facility ratio and the district ratio for three of the variables, ANC1, ANC4, and Penta3, was less than 33% across the eight facilities. Three facilities recorded more than 33% differences between their ratio from the district ratio for IPT1 and Penta1, and two facilities and one facility recorded more than 33% differences between their ratio and the district ratio for Td2 + and PNC on one hand and deliveries on the other hand. There was also a percentage difference of approximately 115% for Penta1, and 73% for Td2 + between one facility’s ratio and district ratio.

**Table 9:** Consistency over time ratios, 2017–2020

Indicator	District ratio	Health Care Facility							
		**A**	**B**	**C**	**D**	**E**	**F**	**G**	**H**
ANC1									
Y	0.91	0.80	1.12	1.17	0.87	0.96	0.77	0.86	1.00
Z	-	12.1	23.1	28.6	4.4	5.5	15.4	5.5	9.9
ANC4									
Y	0.90	0.93	0.82	1.00	0.77	0.83	0.74	.99	1.06
Z	-	3.3	8.9	11.1	14.4	7.8	17.8	10.0	17.8
IPT1									
Y	0.89	0.79	1.21	0.83	0.79	0.51	0.88	1.19	1.02
Z	-	11.2	**36.0**	6.7	11.2	**42.7**	1.1	**33.7**	14.6
Td2 +									
Y	0.79	0.73	0.53	1.02	1.37	0.44	0.65	0.68	0.76
Z	-	7.6	32.9	29.1	**73.4**	**44.3**	17.7	13.9	3.8
Deliveries									
Y	0.94	0.82	1.28	1.20	0.94	0.94	0.78	.95	1.16
Z	-	12.8	**36.2**	27.7	0.0	0.0	17.0	1.1	23.4
PNC									
Y	1.16	0.72	1.01	1.81	0.89	1.54	1.03	.98	1.13
Z	-	**37.9**	12.9	**56.0**	23.3	32.4	11.2	15.5	2.6
Penta1									
Y	0.89	0.84	1.07	1.35	1.91	0.75	0.53	1.02	0.88
Z	-	4.9	20.2	**51.7**	**114.6**	15.7	**40.4**	14.6	1.1
Penta3									
Y	0.97	0.83	1.19	1.11	0.77	1.26	0.72	1.09	0.80
Z	-	13.7	22.7	14.4	20.6	29.9	25.8	12.4	17.5

Y = an indicator’s ratio of 2020 to the mean of the preceding 3 years

Z =  ≥  ± 33% difference from the variables’ district ratio

More than 33% difference between facilities and district ratio are boldened

### Consistency between related data

Internal consistency between variables measures the extent to which the values for two or more variables exhibit a predicted relationship. The variable pairs considered includes: Penta1 and ANC1; Penta1 and Penta3; and ANC1 and ANC4. One facility (facility M) at the time of this study was not providing child health services, hence, only the ANC1 and ANC4 variables pair were analysed for this facility. The ratio of the consistency between the number of Penta1 doses administered and number of ANC1 visit was above 1 in 42% of the facilities showing a higher Penta1 administration than ANC1 coverage (Table 10). The overall ratio of the consistency between the number of Penta1 doses and number of ANC1 visits was 86%.

**Table 10:** Consistency between related variables

Facility	Ratio of variables			Percentage difference		
	ANC4 & ANC1	Penta3 & Penta1	Penta1 & ANC1	ANC4 & ANC1	Penta3 & penta1	Penta1 & ANC1
**A**	0.80	0.52	0.82	-24.44	-92.42	-21.89
**B**	0.80	0.74	1.48	-25.47	-35.99	32.32
**C**	0.77	0.98	0.84	-30.36	-2.50	-65.44
**D**	0.78	0.83	0.60	-28.29	-21.03	-18.7
**E**	0.49	0.41	1.25	-104.63	-143.75	19.76
**F**	0.54	1.67	0.31	-84.62	40.00	-220
**G**	0.59	0.93	0.95	-69.43	-7.25	-5.26
**H**	0.80	0.91	0.54	-24.77	-10.00	-85.18
**I**	0.59	1.16	1.01	-70.00	13.75	1.45
**J**	0.94	1.40	1.89	-6.00	28.57	47
**K**	1.12	1.34	3.06	10.91	25.37	67.33
**L**	0.64	1.25	0.55	-55.78	20.19	-82.35
**M**	1.00	-	-	0	-	
**Overall**	0.70	0.78	0.86			

A 0.78 ratio was observed between the number of Penta1 to Penta3 doses administered. Further, 58% of the facilities had negative percentage difference between the two variables (Penta1 and Penta3), suggesting a higher administration of Penta1 vaccines compared to Penta3. The remaining 42% of the facilities showed lower Penta1 vaccine administration compared with Penta3, as indicated by their positive percentage difference. About 42% of the facilities were observed to have a higher than 2% consistency ratio for these variables (Table 10).

Positive, negative and zero percentage difference were observed in the 13 facilities used in assessing the consistency between ANC1 and ANC4. Specifically, 8% of them had positive and a zero-percentage difference and 92% showed a negative percentage difference. The overall ratio of the consistency between the two variables was 70%.

### Outliers in the reference year

About 1% moderate outliers were detected, and this was observed in the months of May for IPT1, and June for Penta1. None of the MCH variables were prone to extreme outliers (Table 11).

**Table 11:** Consistency of event reporting: outliers in the reference year

Month	ANC1	ANC4	IPT1	Td2 +	Deliveries	PNC Reg	Penta1	Penta3
Jan	560	333	359	244	486	650	361	293
Feb	452	365	388	227	413	510	351	304
Mar	445	365	324	239	587	722	361	288
Apr	399	357	320	183	595	639	371	271
May	336	289	211	174	647	714	398	281
Jun	415	267	271	154	553	547	469	387
Jul	452	278	266	164	587	532	435	327
Aug	445	259	328	151	465	552	399	327
Sept	362	318	347	206	451	575	371	351
Oct	547	343	345	253	508	541	379	305
Nov	548	329	344	264	470	619	438	351
Dec	486	307	341	241	443	585	396	379

Values in bold indicate moderate outliers

## Discussions

This study assessed the level of MCH data quality in the health care facilities at the Cape Coast Metropolis. Data accuracy was assessed by comparing reports (Forms and DHIS2) with source document (registers). The percentage of facility’s MCH data accuracy from the registers to the monthly reporting formats (Forms and DHIS2) was lower than from Forms to the DHIS2. Disparities (over/under reporting) were observed for some of the MCH variables and for the months. However, these disparities were not fatal since the proportion of the reported numbers that were verified from the source documents were within the acceptable tolerance threshold of 100% ± 10% [24] for all the variables, except for Td2 + which was largely under-reported. This suggests that the MCH data transferred from the register to the monthly report forms, register to DHIS2, and forms to DHIS2 were accurate. This finding is similar to a study in Ghana, where newborn health data transferred from facilities registers to the reporting forms and DHIS2 database were reported accurate [25]. As part of performance evaluation in the study area, GHS reemphasizes improvements in MCH service provision [26]. Therefore, under/over reporting services might indicate attempts to claim better performance. It is therefore important to carefully consider these variations (under/over reporting) when using the data for decision making. Underlying these variations is the fact that recording of data into these sources is largely manual and paper-based.

All the government owned facilities reported accurately for all the variables except for deliveries where the data were found to be inaccurate in DHIS2 database. Further, ANC1 and PNC were found to be inaccurate for the private owned facilities. The Teaching Hospital and all the Health Centres had all their MCH data accurate in DHIS2. Whereas clinics and CHPs compounds over reported their PNC services in DHIS2, the Metropolitan hospital hugely under reported its Td2 + services by over two times.

All the healthcare facilities submitted the two monthly reports (Midwife’s Returns and Vaccination Report) on monthly utilization of their MCH services for all the 12 months of 2020, indicating a complete reporting rate. This finding indicates that data generated does not remain at the facility level, but distributed to the next level for necessary action. Sending the reports of the MCH coverage to the next reporting level indicate that the district health offices receive a complete representation of the MCH services provided in their catchment areas. This could have important implications for the health of pregnant women and new-borns, as information reported by the facilities may be used by the officers to guide future plans and inform accomplishments [27]. An assessment of RHIS data in Addis Ababa showed completeness rate of 100% [28], and in Gurage Zone [29], it was found that approximately, 87% of the Primary Health Care Units had a reporting completeness rate of more than 90%. Also, issues of completeness were found in a recent study where 83.3% completeness rate were reported among selected health centers in Southern Ethiopia [30], and 76% completeness rate in data quality assessment performed in Primary Health Care Unit from a total of 17 districts across six regions of Ethiopia [31].

All, except one facility did not meet the set limit for completeness of data in DHIS2 database for the MCH variables. Generally, completeness was best for the Child Health variables, that is., 100% for Penta3, and 99.6% for Penta1, which indicates that all the data were entered into the DHIS2. However, the MCH variables with the most missing values were found in the provision of maternal health services, with observed variations. Further, about 8% of the facilities had 50% of their data in the registers and forms complete. This suggests that the health care professionals are more focused on managing patients rather than recording data, perhaps due to workload or lack of commitment to the data.

In Ghana, DHIS2 is the final repository for data routinely generated from health facilities and is the main source of information used by the majority of the health managers in the country for planning and decision making. Completeness of data in DHIS2 were found to be generally high (95.4%). Assessing the completeness and accuracy of data transfer of routine maternal health services data in the Greater Accra Region of Ghana [11], reported 99.1% completeness in summary reporting forms and 100% in the DHIS2. The authors further reported 94.3% data completeness for the antenatal variables. However, lower completeness rates were found in Nigeria for the Monthly Summary Form at 89.3%, and 65.2% in DHIS2, with an overall average completeness of 77.3% [32]. Relatedly, high (86.9%) data completeness were reported in four counties in Kenya [22], and 96.6% from Rwanda [33]. In contrast, a study in Uasin Gishu County Referral Hospital in Kenya reported as low as 46% routine health data completion [34]. In the analysis of primary health care data in Mozambique, manual data completeness was between 37.5% and 52.1% [35]. The findings of higher completeness rates of data aggregation and transfer in this study could be attributable to a more vigilant process of validating data aggregated from one medium before transferring it to the next [34]. It also shows the availability of qualified human resource, appropriate policies and framework for data management in the metropolis.

The MCH variables completeness for privately owned health facilities (84.8%) was less than public facilities (96.8%). The private sector provides a significant portion of healthcare in developing countries and will contribute significantly to the data available in RHIS [36]. In the past, private healthcare facilities in Ghana did not feel a sense of duty to the government by submitting their routine data. This development is not limited to only Ghana, as other developing countries have reported difficulty in integrating the private and public health information systems [36].

The degree to which data is current and available when needed to make decisions is reflected in its timeliness. Timeliness represents the proportion of reports that are transmitted to the next level of the reporting system within the timeframe stipulated by the GHS. Our findings of timeliness of MCH data is higher than the timeliness reported elsewhere; 70% in East Wollega, Ethiopia [37], 78.7% in four counties in Kenya[22], and 46% reporting timeliness in Uasin Gishu County Referral Hospital, Kenya [22], but similar to studies from Rwanda where 93.8% timeliness was reported [33], and 93.7% timeliness reported among departments in public health facilities of Harari region, Ethiopia [33]. The results revealed that among the two MCH services considered, Monthly Vaccination Report was submitted on time better than Monthly Midwife’s returns. According to [24], facilities are considered to have good reporting if their timeliness rate falls above 80%. Whereas all the facilities met this threshold for the Monthly Vaccination Report, three (23%) facilities did not meet the threshold for Monthly Midwifes Returns report. Meanwhile, timely submission of MCH coverage estimates to the next reporting level is crucial in the provision of MCH services. This would have important implications for the health of pregnant women and newborns living in the district, as information reported by the health facilities is used to guide future plans.

Reported data in DHIS2 for 2020 were consistent for all the eight MCH variables in the Metropolis. Consistency over time indicated an overall 7% decrease in the MCH service outputs for 2020 when compared with that of the preceding three years across the eight variables. Apart from PNC that showed a ratio of over 100%, the rest of the variables were below 100%. Nevertheless, all the variables remained within the quality range of 33% of the average for the three preceding years. Therefore, facilities in this study were more likely to report consistently in 2020, compared to the preceding three years for all the MCH variables. All the facilities data for ANC1, ANC4 and Penta3 were consistent over time. However, some of the variables and facilities data were found not consistent, when a facility data for a variable was compared to that of the district value. It is generally impracticable to have same values of an indicator over a period of time. Changes are expected in the current year to the preceding year(s), but these variations are mostly expected not to be large. If the differences are so large, it calls for concern and raises issues of data quality.

The overall ratio of the consistency between the number of Penta1 doses administered to children and number of ANC1 visits was low (86%). This means that, roughly 14% more women attended ANC1 visit than children receiving their first dose of Penta, or that there were data quality challenges. This variation may also reflect a higher number of pregnancies than live births, which was not assessed directly in this study. Typically, women accessing health care during pregnancy have at least one ANC visit to the health facility and that most children that seek health care in their first year of life will have at least one visit to the health facility. In fact, evidence has shown that women who seek ANC services are more inclined to seek health services and the essential vaccinations such as Pentavalent vaccine for their newborns [24]. The inconsistency between Penta1 and ANC1 presents potential gaps that warrant further investigation and raise concerns for data quality.

Comparing the number of Penta1 to Penta3 doses administered, it was observed that about 22% of the children who received the first dose of Penta vaccine did not receive the third dose. Further, 42% of the facilities showed lower Penta1 vaccine administration compared with Penta3, as indicated by their positive percentage difference. The finding suggests that many infants who received their third dose may not have received their first dose in these facilities, an issue that warrants further investigation. Accordingly, the percentage difference of the number of Penta3 dose and Penta1 dose should be less than two percent for data between the indicator pairs to be consistent [24]. About 42% of the facilities were observed to have a higher than two percent consistency ratio for these variables. Generally, the number of Penta1 doses should be either more than Penta3 or be the same. However, there is the possibility, theoretically, that the number of third dose of Penta is slightly more than the first, especially for administrative units with a lot of in-migration, but it is not likely to happen systematically [24].

Of the 13 facilities used in assessing the consistency between ANC1 and ANC4, only one showed a positive percentage difference, suggesting higher a ANC4 uptake compared with ANC1. Higher coverage of ANC4 to ANC1 may be indicative of data quality limitation because it is expected that ANC1 would be higher than ANC4 coverage [24]. Also, 8% of the facilities showed a zero-percentage difference between the two variables which suggests that the same number of pregnant women who attended first ANC visit also attended the fourth ANC visit in that same facility. The overall ratio of the consistency between the two variables was 70%, indicating that 30% of pregnant women who attended the ANC1 did not attend ANC4 visit. Across all facilities, none of the priority variables compared demonstrated the expected numerical relationship.

Access to data has increased nowadays due to technological advancement, but the quality of data has been identified as critical area needing intervention. Meanwhile, quality data is essential for monitoring and evaluating MCH services to improve health outcomes [38]. A number of factors can be attributable to variations in data from one source to another. For example, incomplete source documents and errors in computation when aggregating data could lead to over-reporting of data from registers to monthly reported forms. Previous studies identified insufficient time due to workload, lack of appreciation of the importance of data, transcription errors, and transposing errors [11]. Generally, data accuracy may be affected by errors that occur during data entry, intentionally manipulating the data for different reasons, possibly competition among the staff and facilities, false report to increase achievement, and reports not made on time. The study conducted in Tanzania supports some of these explanations; for example, data manipulation can affect the accuracy of data [14]. In Ghana, most health facilities especially in the lower levels, use lower cadre of staff who do not have the requisite training in data management [11]. However, in this current study, majority of the respondents had higher education.

### Limitations

Primary source data was used as reference for comparing the other sources of data. Any shortcoming in capturing data into these primary sources will therefore reflect in our results. The study was a stand-alone survey and therefore is limited in linking facility data with community service utilization. The study did not consider the external consistency of the data by comparing it to any population metrics, as well as not considering variables beyond MCH services. Lastly, although this study is important and relevant for Cape Coast, this research likely has limited external validity beyond Cape Coast Metropolis.

## Conclusion

Large variability of data accuracy was observed across the facilities. This is an important concern in data quality as it may prevent comparisons between facilities and the understanding of inequalities in healthcare quality in the region. Notwithstanding, given the WHO standard for data quality, the level of MCH data quality in the Cape Coast Metropolis, available through the DHIS2 is complete, reported on timely manner, consistent, and reflects what exists in facility’s source document (registers). Therefore, the data is credible enough for day-to-day use in health decision making, even though there is still room for improvement in the quality of these data, especially when the data is compared across facilities.

Maternal and child health services cannot be adequately monitored where health information data are inaccurate, incomplete, untimely, or inconsistent [39]. Decisions made based on inaccurate data can affect health system performance and consequently mislead directions. Therefore, system should design strategies and be watchful to maintain data quality. Data validation teams in the various health facilities should be encouraged to have their data validated before transmitting the data to the next level. Consistent use of the Standards Operating Procedures (SOPs) for data management in the metropolis should be greatly encouraged.

## Data Availability

The datasets generated and/or analysed during the current study are part of the corresponding author’s ongoing PhD thesis, and hence, are not publicly available, but are available from the corresponding author on reasonable request.

## Abbreviations

ANC4: Antenatal Care (visit) 4
CI: Confidence Interval
DHIS2: District Health Information System
IPT1: Intermittent Preventive Treatment in Pregnancy first dose
MCH: Maternal and Child Health
Penta1: Pentavalent Vaccine first dose
Penta3: Pentavalent Vaccine third dose
PNC: Postnatal Care
RHIS: Routine Health Management Information System
SPSS: Statistical Package for the Social Sciences
Td2 +: Pregnant women receiving at least the 2nd dose of Tetanus–Diphtheria Vaccine
WHO: World Health Organisation

## References

[1] United Nations (2015). Transforming our world: the 2030 agenda for sustainable development.

[2] Farnham A, Utzinger J, Kulinkina AV, Winkler MS (2020). Using district health information to monitor sustainable development. Bull World Health Organ.

[3] Hotchkiss DR, Diana ML, Foreit KGF (2012). How can routine health information systems improve health systems functioning in low-and middle-income countries? Assessing the evidence base. Health Inf Technol Int Context..

[4] World Health Organization (2017). Data quality review: module 1: framework and metrics.

[5] AbouZahr C, Boerma T (2005). Health information systems: the foundations of public health. Bull World Health Organ.

[6] Mucee EM, Kaburi LW, Kinyamu RK (2016). Routine health management information use in the public health sector in Tharaka Nithi County, Kenya. Imperial J Interdisciplinary Res.

[7] Nisingizwe MP, Iyer HS, Gashayija M, Hirschhorn LR, Amoroso C, Wilson R (2014). Toward utilization of data for program management and evaluation: quality assessment of five years of health management information system data in Rwanda. Glob Health Action.

[8] Arsenault C, Gage A, Kim MK, Kapoor NR, Akweongo P, Amponsah F, et al. COVID-19 and resilience of healthcare systems in ten countries. *Nature Medicine*. 2022:1–11. 10.1038/s41591-022-01750-1 PMC9205770 35288697

[9] Hung YW, Hoxha K, Irwin BR, Law MR, Grépin KA (2020). Using routine health information data for research in low- and middle-income countries: A systematic review. BMC Health Serv Res.

[10] Doubova SV, Leslie HH, Kruk ME, Pérez-Cuevas R, Arsenault C (2021). Disruption in essential health services in Mexico during COVID-19: An interrupted time series analysis of health information system data. BMJ Glob Heal.

[11] Amoakoh-Coleman M, Kayode GA, Brown-Davies C, Agyepong IA, Grobbee DE, Klipstein-Grobusch K (2015). Completeness and accuracy of data transfer of routine maternal health services data in the greater Accra region. BMC Res Notes.

[12] Bhattacharya AA, Umar N, Audu A, Felix H, Allen E, Schellenberg JRM (2019). Quality of routine facility data for monitoring priority maternal and newborn indicators in DHIS2: a case study from Gombe state. Nigeria PLoS One.

[13] Endriyas M, Alano A, Mekonnen E, Ayele S, Kelaye T, Shiferaw M (2019). Understanding performance data: health management information system data accuracy in Southern Nations Nationalities and People’s Region, Ethiopia. BMC Heal Serv Res.

[14] Rumisha SF, Lyimo EP, Mremi IR, Tungu PK, Mwingira VS, Mbata D (2020). Data quality of the routine health management information system at the primary healthcare facility and district levels in Tanzania. BMC Med Informatics.

[15] Teklegiorgis K, Tadesse K, Terefe W, Mirutse G (2016). Level of data quality from Health Management Information Systems in a resources limited setting and its associated factors, eastern Ethiopia. South African J Inf Manag.

[16] Ouedraogo MO. Maternal and Child Health in Jimma Zone, Ethiopia: Predictors, Barriers and Strategies for Improvement (Doctoral dissertation, Université d'Ottawa/University of Ottawa). 2018.

[17] Chen H, Hailey D, Wang N, Yu P (2014). A review of data quality assessment methods for public health information systems. Int J Environ Res Public Health.

[18] Fox C, Levitin A, Redman T (1994). The notion of data and its quality dimensions. Inf Process Manag.

[19] Nshimyiryo A, Kirk CM, Sauer SM, Ntawuyirusha E, Muhire A, Sayinzoga F (2020). Health management information system (HMIS) data verification: a case study in four districts in Rwanda. PLoS ONE.

[20] Ouedraogo M, Kurji J, Abebe L, Labonté R, Morankar S, Bedru KH (2019). A quality assessment of health management information system (HMIS) data for maternal and child health in Jimma zone. Ethiopia PloS one.

[21] Maïga A, Jiwani SS, Mutua MK, Porth TA, Taylor CM, Asiki G (2019). Generating statistics from health facility data: the state of routine health information systems in eastern and southern Africa. BMJ Glob Heal.

[22] Manya A, Nielsen P. Reporting practices and data quality in health information systems in developing countries: an exploratory case study in Kenya. J Heal Informatics Dev Countries. 2016;10:114-26.

[23] Ghana Health Service. 2010 population and housing census: Summary report of final results. 2012;1:1–117.

[24] World Health Organisation (2014). Guide to the health facility data quality report card: World Health Organisation.

[25] Achampong EK, Adzakpah G, Boadu RO, Lasim O. The Quality of Newborn Data: Assessment of Data Management and Reporting System. Int J Public Heal Sci. 2018;7:194-200.

[26] Ghana Health Service (2014). Annual Report.

[27] Bhattacharyya S, Berhanu D, Taddesse N, Srivastava A, Wickremasinghe D, Schellenberg J (2016). District decision-making for health in low-income settings: a case study of the potential of public and private sector data in India and Ethiopia. Heal Policy Plan.

[28] Tadesse K, Gebeyoh E, Tadesse G (2014). Assessment of health management information system implementation in Ayder referral hospital, Mekelle. Ethiopia Int J Intell Inf Syst.

[29] Tsedeke M. Community health management information system Performance and factors associated with at health post of Gurage zone, SNNPR, Ethiopia [Internet]. M.Sc. Thesis, University of Gondar and Addis Continental Institute of Public Health; 2015.

[30] Solomon M, Addise M, Tassew B, Balcha B, Abebe A (2021). Data quality assessment and associated factors in the health management information system among health centers of Southern Ethiopia. PLoS ONE.

[31] Gebrekidan M, Hajira M, Habtamu T, Negusu W, Dereje M, Nafo-Traoré F (2012). Data quality and information use: A systematic review to improve evidence. Ethiop Int J Intell Inf Syst.

[32] Adejumo A (2017). An assessment of data quality in routine health information systems in Oyo State, Nigeria.

[33] Karengera I, Anguyo R, Onzima DDM, Katongole SP, Govule P (2016). Quality and use of routine healthcare data in selected districts of eastern province of Rwanda.

[34] Cheburet SK, Odhiambo-Otieno GW. State of data quality of routing Health Management Information System: Case of Uasin Gishu County Referral Hospital, Kenya. Int Res J Public Environ Heal. 2016;3:174-81.

[35] Gimbel S, Micek M, Lambdin B, Lara J, Karagianis M, Cuembelo F (2011). An assessment of routine primary care health information system data quality in Sofala Province. Mozambique Popul Heal Metrics.

[36] Berman P, Rose L (1996). The role of private providers in maternal and child health and family planning services in developing countries. Health Policy Plan.

[37] Fikru ND, Dereje BD (2018). Evaluation of HMIS data quality and information use improvement for local action-oriented performance monitoring in Beghi District in West Wollega, Oromia. Ethiopia Heal Med Nurs.

[38] Lucyk K, Tang K, Quan H (2017). Barriers to data quality resulting from the process of coding health information to administrative data: a qualitative study. BMC Heal Serv Res.

[39] Mutale W, Chintu N, Amoroso C, Awoonor-Williams K, Phillips J, Baynes C (2013). Improving health information systems for decision making across five sub-Saharan African countries: implementation strategies from the African Health Initiative. BMC Heal Serv Res.

